# Research progress in intratumoral microbiota: unlocking the potential of cancer immunotherapy

**DOI:** 10.3389/fimmu.2026.1956198

**Published:** 2026-10-01

**Authors:** Tian Tian, Min Chen

**Affiliations:** Department of Critical Care Medicine, Youxian People’s Hospital, Zhuzhou, China

**Keywords:** cancer treatment, immunotherapy, intratumoral microbiota, targeted therapy, tumor immune microenvironment

## Abstract

In recent years, intratumoral microbiota (ITM), a crucial component of the tumor microenvironment, have received increasing attention because of their role in cancer immunotherapy. ITM not only influence tumor initiation and progression but also modulate local and systemic immune responses, thereby affecting the efficacy of immune checkpoint inhibitors and other immunotherapeutic approaches. This review summarizes the compositional characteristics of ITM, their immunoregulatory mechanisms, and their effects on tumor metastasis and responsiveness to immunotherapy. Particular emphasis is placed on interactions between intratumoral microbiota and immune therapies across cancer types, highlighting potential therapeutic strategies that exploit these microbial influences. Furthermore, the review discusses current challenges in integrating intratumoral microbiota into immunotherapeutic paradigms and outlines future research directions. By providing a comprehensive overview of the role of ITM in cancer immunity, this review aims to support advances in precision oncology and foster novel approaches to enhance immunotherapy outcomes.

## 1 Introduction

The human body is estimated to harbor approximately 38 trillion microorganisms, which contribute to the regulation of physiological processes and susceptibility to disease (1). Intratumoral microbiota (ITM) comprise microbial communities residing within tumor tissues, including bacteria, fungi, viruses, and mycoplasmas (2, 3). Although microorganisms were traditionally recognized in body cavities and organs exposed to the external environment (e.g., gut, skin, oral cavity, and vagina) (4), technological advances have revealed low-biomass microbial communities in tissues and organs previously considered sterile, including the lung, breast, liver, pancreas, prostate, and kidney (5, 6). These findings led to the concept of ITM, defined as microorganisms residing within tumor tissues (7). ITM may originate from the local microbiota of the tumor-bearing organ or translocate from distant sites, such as the gut. This polymorphic microbiome has been proposed as a novel hallmark of cancer because of its profound effects on tumor initiation, progression, and metastasis (8). The tumor microenvironment (TME), with its complex and diverse composition and functions, is a major focus of contemporary cancer biology and treatment research. As an important component of the TME, ITM are increasingly recognized as key regulators of tumorigenesis, progression, and treatment response. The composition and selective colonization of ITM are shaped by the distinctive features of the TME, which is often hypoxic, immunosuppressive, and highly permeable and therefore provides niches that favor specific microbial populations (9). Importantly, ITM modulates the mutational landscape and gene function of tumor cells and orchestrates the immune milieu by regulating inflammation, angiogenesis, and the stem cell niche, thereby remodeling the tumor immune microenvironment (TIME) (10).

Immunotherapy, particularly the widespread use of immune checkpoint inhibitors (ICIs), has markedly improved outcomes in several cancers and represents a major milestone in cancer treatment. However, many patients continue to experience low response rates and primary or acquired resistance, which limit the broader clinical application of immunotherapy (11). Growing evidence indicates that interactions between the microbiota within the TME and the immune system are key determinants of immunotherapy efficacy (12, 13). Microorganisms colonizing tumor tissues can contribute to immune escape and inflammatory responses and regulate the recruitment and activation of immune cells through direct or indirect immunomodulatory mechanisms, thereby shaping the TIME and influencing immunotherapy efficacy (14, 15). Large-scale research initiatives have therefore been undertaken to characterize the relationships between ITM and cancer (**Figure 1**).

**Figure 1:**
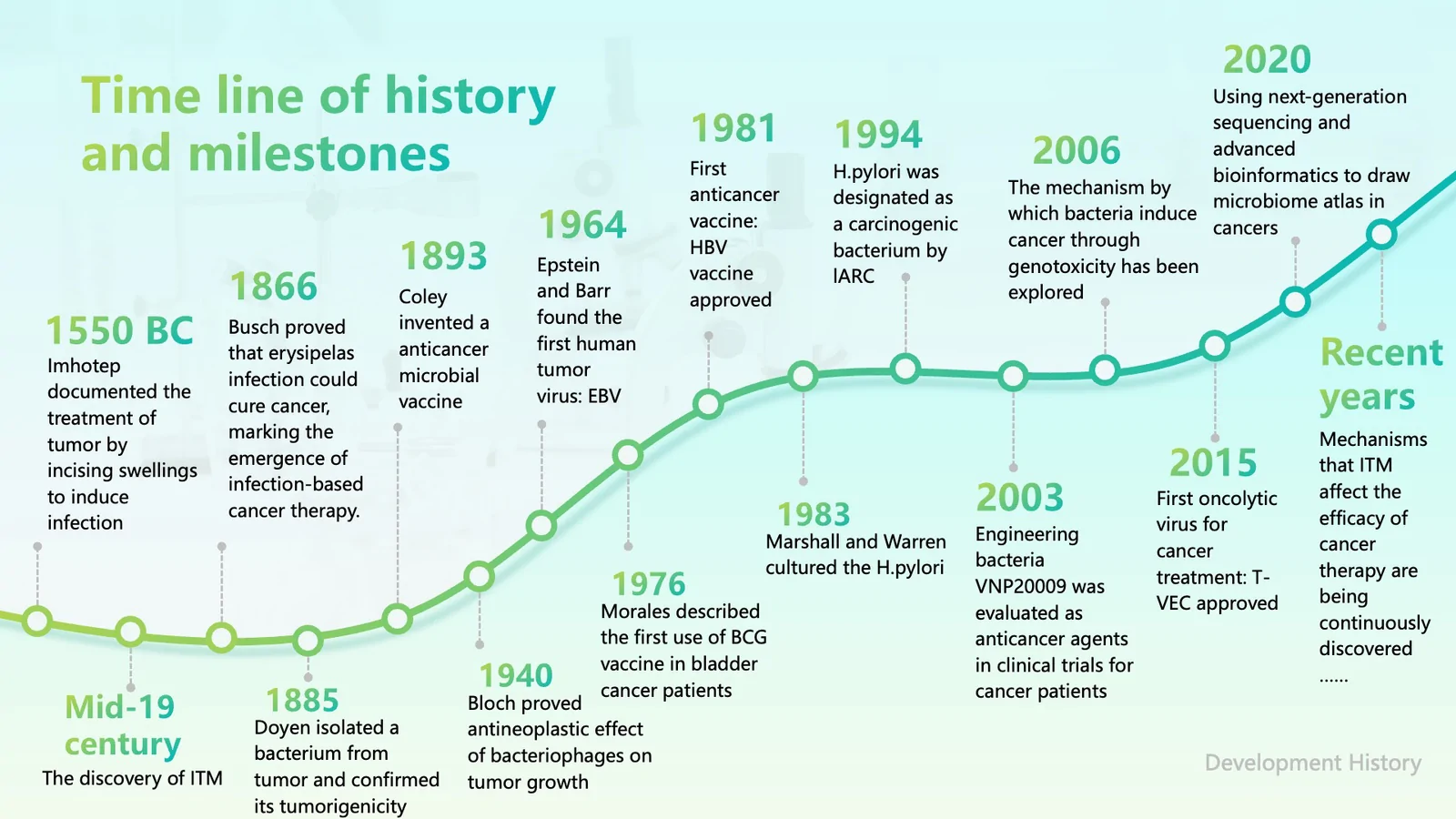
Schematic overview of the development of intratumoral microbiota research.

Recent studies have increasingly focused on the interactions between ITM and immunotherapy and their potential clinical applications. Elucidating how tumor-associated microbes regulate immunity and therapeutic responses may provide new opportunities to harness the microbiome for precision oncology. This review systematically summarizes current knowledge of the composition and functions of ITM, elucidates its regulatory mechanisms and crosstalk with immunotherapies, and discusses the clinical prospects and challenges of microbiome-targeted strategies for realizing the full potential of cancer immunotherapy.

## 2 Tumor-resident bacteria mechanisms and intratumoral microbial dynamics

### 2.1 Composition and spatial heterogemen of ITM

The reason why tumor tissues become ideal colonization sites for microbiota, especially for bacteria, is mainly due to the following reasons: Firstly, Abnormal tumor angiogenesis generates vascular defects enabling microbial infiltration (16, 17). Secondly, tumors can suppress the immune response through multiple mechanisms, creating an “immune privilege” state that reduces the attack of immune cells on them (18), which also provides microorganisms with an environment that is less threatened by the host’s immune system clearance. Finally, intratumoral hypoxia together with necrotic areas and substances produced by metabolic activities supplies nutrients supporting facultative and obligate anaerobe proliferation (19). Due to these structural and functional characteristics, tumor tissues have become an attractive colonization site for a variety of microorganisms.

However, microbial profiles differ across cancer types and vary within discrete regions of a single tumor: 1) The abundance is far lower than that in the intestinal tract (20); 2) The diversity of microbial communities within tumors is usually lower than that in normal tissues (21); 3) Livyatan et al. proved that ITM can be mostly inside both tumor and immune cells rather than in the extracellular compartment. To verify that tumors harbor viable bacteria rather than merely bacterial debris, they demonstrated that bacteria can be cultured from the tumors tissues (22). Generally speaking, ITM denotes microbial communities colonizing tumor tissues and exhibits remarkable spatial heterogeneity, which is manifested by variations in microbial composition and abundance across different cancer types as well as uneven microbial distribution within distinct regions of the same tumor (**Table 1**). It’s worth noting that ITM and gut microbiota represent two distinct microbial communities. The gut microbiota colonizes the intestinal lumen and mucosal surfaces, featuring high biomass and participates in host nutrient metabolism, intestinal barrier maintenance and systemic immune regulation. In contrast, ITM resides within tumor tissues and belongs to low-biomass microbial populations with much lower microbial load compared with the gut. Gut microbiota remotely modulate tumor progression mainly via circulating metabolites and immune-cell migration. In contrast, ITM directly infiltrate the tumor immune microenvironment, locally regulating malignant phenotypes of tumor cells and *in-situ* immune responses. These two microbial compartments are not entirely independent, as certain microorganisms can translocate from the gut to tumors. Although ITM are distinct from the intestinal microbiota, both microbial communities can shape immunotherapy responses by regulating the TME and immune-cell function. Besides, gut microbiota-associated systemic effects should not be equated with direct colonization of tumor tissue; evidence for a functional effect of the gut microbiome on immunotherapy does not, by itself, establish the presence or activity of the same microorganisms within the tumor.

**Table 1:** Microbial heterogeneity in different tumors.

Tumor type	Microbiome compositions	Abundance dynamics	Function	Ref
Oral squamous cell carcinoma	*Parvimonas, Peptoniphilus, Fusobacterium*	↑	Reduces the protein expression of the tumor suppressor p53	(23)
	*Eubacterium infirmum, Actinobaculum, Selenomas*	↓	immune therapy resistance	(24)
	*Porphyromonas gingivalis*	↑	Related to the formation of DNA adducts or the inhibition of DNA repair enzymes; Related to EMT-related transcription factors;	(25)
	*Prevotella intermedia*	↑	Related to carcinogenesis;	(26)
	*Treponema denticola*	↑		(27)
	*Streptococcus anginosus*	↑	Related to the formation of DNA adducts or the inhibition of DNA repair enzymes;	(28)
	*Streptococcus*	↓		(29)
	*Pseudomonas aeruginosa, Campylobacter* sp. *Oral taxon 44*	↑		(30)
	*Human papillomavirus*	↑		(31)
	*Epstein-Barr virus*	↑		(32)
	*Herpes Simplex Virus Type 1*	↑		(33)
	*Candida albicans, Candida etchellsii, Hannaella luteola-like*	↑		(34)
Head-and-neck squamous cell carcinomas	*Parvimonas*	↑		(35)
	*Actinomyces*	↓		(35)
	*Human papillomavirus types 16*	↑	Inhibited the cGas-STING pathway;	(36)
Nasopharyngeal carcinoma	*Corynebacterium, Staphylococcus*	↑		(37)
	*Epstein-Barr virus*	↑	Downregulated IDO expression;	(38)
Colorectal cancer	*Fusobacterium, Bacteroides*	↑	Activates the MAPK signaling pathway	(23)
	*Campylobacter, Peptostreptococcus*	↑	tumor progression	(39)
	*Candida*	↑	tumor progression	(40)
	*Malassezia, Aspergillus*	↑	tumor high KRAS and MSI mutation	(41)
	*Enterotoxigenic Bacteroides fragilis*	↑	Secreted carcinogenic toxins; initiated pro-inflammatory signaling cascade;	(42)
	*Fusobacterium*	↑	Promoted the polarization of M2-like macrophages	(43)
	*Lactococcus, Bacteroides, Prevotella, Streptococcus*	↑		(44)
	*Pseudomonas, Escherichia-Shigella*	↓		
	*Bifidobacterium, Romboutsia*	↑	Related to left-sided colon cancers	(45)
	*Haemophilus, Veillonella*	↑	Related to right-sided colon cancers	(45)
	*Ascomycota, Malasseziomycetes*	↑		(46)
	*Saccharomycetes, Pneumocystidomycetes*	↑		(46)
	*Escherichia coli*	↑	Secreted carcinogenic toxins, related to metastasis	(47)
	*Enteropathogenic Escherichia coli*	↑	Disrupted mechanisms of DNA mismatch repair;	(48)
	*Hungatella hathewayi*		Upregulated DNA methyltransferases	(49)
	*Akkermansia*		Increased IL-17 production and B cell infiltration	(50)
	*Candida*	↑	Involved in the downregulation of genes mediating cellular adhesion;	(51)
Breast cancer	*Methylobacterium radiotolerans*	↑	Promotes tumor progression	(52)
	*Bacillus, Enterobacteriaceae, Staphylococcus, Staphylococcus epidermidis*	↑	Related to tumor progression; Enhanced the resistance of tumor cells to flow shear stress;Caused double-stranded DNA breaks;	(53)
	*Pseudomonas, Proteus*	↑		(54)
	*Sphingomonas yanoikuyae*	↓		(55)
	*Cladosporium*	↑		(3)
	*Lactobacillus*	↑	Metabolic regulation; enhanced the resistance of tumor cells to flow shear stress;	(56)
	*Fusobacterium, Atopobium, Hydrogenophaga, Gluconacetobacter*	↑	Metabolic regulation;	(57)
	*Bosea*	↑	Related to tumor progression;	(58)
	*Enterotoxigenic Bacteroides fragilis*	↑	Enhanced stemness potential and metastatic progression;	(17)
	*Fusobacterium nucleatum*	↑	Related to metastasis;	(59)
Pancreatic ductal adenocarcinoma	*Gammaproteobacteria*	↑	Mediates response to gemcitabine-based chemotherapy	(60)
	*Pseudoxanthomonas, Streptomyces, Saccharopolyspora, Bacillus clausii*	↑	Induces an antitumor response and activation of the immune system	(61)
	*Malassezia*	↑	Inhibits tumor growth	(62)
	*Proteobacteria*	↑		(63)
	*Helicobacter pylori*	↑	Related to activation of molecular pathways for tumor growth and progression;	(64)
	*Elizabethkingia*	↑		(63)
	*Acinetobacter, Sphingopyxis*	↑	Related to carcinogenesis;	(65)
lung cancer	*Propionibacterium, Enterobacteriaceae*	↓		(66)
	*Modestobacter*	↑		(66)
	*Blastomyces*	↑		(51)
	*Agaricomycetes, Aspergillus*	↑		(3)
	*Acidovorax*	↑		(67)
	*Klebsiella, Anaerococcus*	↑		(67)
	*Acinetobacter, Brevundimonas, Propionibacterium*	↑		(68)
	*Cyanobacteria*	↑		(69)
	*Veillonella, Megasphaera*	↑	As the diagnostic biomarker of tumor	(70)
	*Family Coriobacteriaceae, genus Pasteurella*	—	Related to the number of CD8 + T cells and M2 macrophages	(71)
	*Nontypeable Haemophilus influenzae*	↑	Released IL-17C and recruited the neutrophils	(72)
liver cancer	*Hepatitis B virus*	↑	Integrated viral genome into the host chromosome; Induced m6A modification of RNA Recruited Treg cells	(73)
	*Hepatitis C virus*	↑	Recruited Treg cells	(74)
	*Helicobacter pylori*	↑		(75)
	*Gammaproteobacteria*	↑		(76)
	*Streptococcaceae, Lactococcus*	↑	As the hallmark groups of cirrhosis hepatocellular carcinoma	(76)
	*Enterobacteriaceae*	↑		(77)
	*Caulobacteraceae, Rickettsiaceae*	↓		(77)
	*Paraburkholderia fungorum*	↓	Related to antitumor activity	(78)
gastric cancer	*Prevotella, Streptococcus, Veillonella, Haemophilus, Neisseria*	↑		(79)
	*Helicobacter pylori*	↑	Related to early-stage of tumors; disrupted DNA mismatch repair mechanisms; inducted abnormal DNA methylation; activated CEACAM1 on immune cells; upregulated PD-L1 expression;	(80)
	*Lactobacillus*	↑	tumor progression	(81)
	*Nitrospirae, Lachnospiraceae, Escherichia-Shigella, Burkholderia fungorum*	↑		(82)
	*Oceanobacter, Methylobacterium, Syntrophomonas*	↑		(83)
	*Propionibacterium acnes, Prevotella copri*	↑	Related to early-stage of tumors	(84)
	*Porphyromonas, Neisseria, Streptococcus sinensis*	↓	Related to benign gastric disease;	(85)
	*Lachnospiraceae, Lactobacillus coleohominis*	↑	Related to malignant gastric disease;	(85)
	*Epstein-Barr virus*	↑	Related to DNA hypermethylation;	(86)
	*Clostridium, Comamonadaceae, Moryella, Vibro, Paludibacter, Agrobacterium*	↑	Monitor the risk of gastric cancer development;	(87)
	*Kytococcus sedentarius, Actinomyces oris*	↑	Inducted abnormal DNA methylation	(88)
Ovarian carcinoma	*Aquificae, Planctomycetes*	↑		(89)
	*Crenarchaeota*	↓		(89)
	*Human papillomavirus types 16, 18, and 45*	↓	Related to advanced-stage tumors;	(90)
			
Endometrial cancer	*Bacteroides, Faecalibacterium*	↑		(91)
	*Staphylococcus, Blautia, Parabacteroides*	↓		(91)
			
Cervical cancer	*Gardnerella, Prevotella, Streptococcus, Atopobium*	↑	Metabolic regulation;	(92)
	*Human papilloma virus*	↑	Integrated viral genome into the host chromosome; recruited Treg cells; inhibited cytotoxic T and NK cell activation;	(92)
Prostatic cancer	*Cutibacterium acnes*	↑		(93)
	*Staphylococcus aureus*	↑	Recruited Treg cells;	(94)
Bladder cancer	*Escherichia coli, Butyrate-producing bacteria SM4/1*,	↑	Related to the expression of EMT-related genes;	(95)
Pituitary neuroendocrine tumor	*Clostridiales, Fusobacteriaceae, Tissierellaceae, Aerococcaceae, Corynebacteriaceae*,	↑	Related to different clinical phenotypes of tumor;	(96)
Hodgkin’s lymphoma	*Epstein-Barr virus*	↑	—	(97)

↑, increase; ↓, decrease.

Importantly, bacteria tend to localize in microniches characterized by distinct immune and metabolic profiles, influencing the TIME and modulating therapeutic responses (23, 98), as exemplified by *Fusobacterium nucleatum* preferentially localizing to tumor-cell-rich regions in breast cancer(BC) (99). A large multicenter cohort study found that a wide variety of intratumoral fungi are widely present in tumors, and these are related to the intratumoral bacterial community (3). For instance, *Malassezia* spp. can translocate from the intestinal lumen into tumors for colonization in patients with pancreatic cancer. Their cell-wall polysaccharides bind mannose-binding lectin to activate the complement cascade, thereby facilitating pancreatic cancer progression (62). By contrast, resident fungus *Aspergillus sydowii* in lung tissues activates the Dectin-1/CARD9 signaling pathway in macrophages via β-glucan, triggering IL-1β production to induce MDSC differentiation. This suppresses the cytotoxic activity of killer T cells and drives CD8^+^ T-cell exhaustion (100). As for the virus of ITM, a key oncogenic factor of EBV, the non-coding RNA BART has been demonstrated to target the apoptosis-associated gene PUMA. It suppresses MHC-I expression in tumor cells, blunts CD8^+^ T-cell responses against EBV-infected cells, and consequently promotes tumor initiation and progression (101, 102). HPV represents another prevalent oncogenic virus that infects cutaneous epithelial cells, as well as oral and genital mucosal cells. The HPV-encoded E6 protein reduces the activities of intracellular antioxidant enzymes SOD2 and GPx, elevating intracellular ROS production and triggering oxidative stress, which further remodels the tumor immune microenvironment (103, 104).

Multiple factors drive ITM spatial heterogeneity: Firstly, the anatomical location of tumors and local microenvironments serve as key driving factors. Gastrointestinal tumors exhibit a high degree of similarity between ITM and gut microbiota owing to direct contact with luminal microbiota (105), while oral microbes can also colonize distant tumors via hematogenous dissemination. For example, *F. nucleatum*, has been found in OSCC, gut cancers, and BC, can colonize the local cancer through circulation and promote carcinogenesis process by inhibiting the immune response directly (106–108). Hypoxic-acidic niches and pH-taxis mediate strain-specific enrichment to exert pro-carcinogenic effects. In other words, the microbial composition varies heterogeneously across different regions within the same tumor owing to the local microenvironment subsection (109). Furthermore, the bidirectional crosstalk between the immune system and microbiota is also critical: immune escape by tumor cells facilitates bacterial colonization, and microbes can shape immune-desert regions by secreting immunosuppressive factors and recruiting immunosuppressive cells (110). Galeano et al. found that ITM mainly reside in high immunosuppression (23). In turn, the distribution of immune cells modulates microbial colonization and spatial distribution (111). Recent researches showed that intratumoral *F. nucleatum* activates NF-κB signaling to facilitate gastric cancer immune evasion by promoting tumor-associated neutrophil recruitment that sensitizes tumors to immune checkpoint blockade(ICB) therapy (112). In addition, intermicrobial interactions (e.g., competitive and symbiotic relationships) and metabolic synergy alter the overall profile of ITM. *Proteobacteria* were predominant in adenomas at first, but replaced by *Firmicutes* along with tumor progression (113), which was exhibiting a mutually exclusive relationship between both microorganisms. While the abundances of *Bacteroides* and *Parvimonas* both increase in CRC, showing a symbiotic relationship. What’s more, mounting evidence indicates that specific bacteria engage in the modulation of amino acid metabolic pathways within tumor cells, a process that promotes their site-specific enrichment in the tumor niche (114, 115). Therapeutic interventions such as chemotherapy and antibiotic treatment disrupt TME and microbial community structure, thereby further exacerbating the spatial heterogeneity of ITM and potentially compromising therapeutic efficacy.

### 2.2 Detection, identification techniques and functional analysis of ITM

Detection, identification and functional analysis constitute core pillars of ITM research. Characterizing ITM encounters multiple obstacles: low microbial biomass, insensitive culture workflows, high risk of exogenous contamination, and limited performance of conventional 16S rRNA sequencing for low-biomass specimens (116). Nevertheless, detection, identification and analytical approaches in this field have advanced rapidly in recent years.

#### 2.2.1 Detection and analysis techniques

Fluorescence in-situ hybridization (FISH), immunohistochemistry (IHC) and correlative light-electron microscopy (CLEM) are employed to localize ITM (117). FISH is restricted by cross-interference of fluorescent signals, limiting the number of applicable probes (118). IHC images microbiota via enzyme- or fluorophore-conjugated specific antibodies, yet fluorophore spectral overlap hampers its performance in complex tissue samples (119). By combining advantages of light and electron microscopy, CLEM uses fluorescent probes for in-situ, high-resolution precise localization of microbes, representing a promising tool for ITM analysis (120). Therefore, it is a useful way to apply FISH and IHC for preliminary localization first and further confirm the predominantly intracellular distribution of ITM with CLEM.

For low-biomass sequencing, accurate identification via high-throughput approaches is of great significance. Metagenomic sequencing performs high-throughput sequencing of all microbial DNA in samples. Following sequence processing and analysis, it can resolve taxonomic composition, phylogenetic relationships, and even functional profiles of microbial communities (121). However, owing to the contamination and low-biomass of ITM in tumor tissues, metagenomic sequencing is poorly suited for characterizing ITM. Recently, researchers have applied a novel PCR method amplifying five regions (5 R) across the bacterial 16S rRNA gene (22, 117). This technique conducts multiplex-PCR amplification and sequencing across five regions (V2, V3, V5, V6, V8) of the 16S rRNA gene. Compared with conventional 16S rRNA amplicon sequencing, this multi-region strategy substantially improves taxonomic coverage and resolution, and is especially suitable for low-biomass microbial samples (122). Besides, Galeano (23) has developed a single-cell RNA sequencing method: invasion-adhesion directed expression sequencing technology, which can better study the interaction mechanism between ITM and tumor tissues. By applying it, ITMs and their interacting host cells can be identified, and the alterations in transcriptional pathways involved in inflammation, metastasis, cell dormancy and DNA repair can be revealed. This method enables the analysis of two types of transcriptome information of tumor cells and microbiota at the single-cell level (123). By the way, The TCGA database provides RNA-SEq and NGS data of thousands of cases in cancers, including gene fragment information of the microbiota in tumor tissues.

#### 2.2.2 Culture approaches

Owing to the low biomass and other difficulties that the corresponding culture methods are not sensitive enough and the operation process is prone to external contamination, methods that are independent from pure culture like metabolomics, genomics and transcriptomics are widely used to speculate potential functions and mechanisms of intratumor microbes. However, for exploring function features of ITM and gaining results of *in vitro* and *in vivo* experiments based on the culture of those microorganisms, pure culture isolation of ITM is still needed. Cultivation serves as a critical complementary strategy to metagenomics, enabling the characterization of previously undescribed microbial taxa (124). Lagier et al. (125) used the methods and conditions that mimic the natural environment for culturing microbes, and this approach led to the successful culture of *Candidatus Pelagibacter ubique* and many other microbes. Based on this strategy, Jalili-Firoozinezhad and colleagues established a microfluidic intestinal organ chip platform, which supported the successful cultivation of bacterial species spanning 11 distinct genera (126). Reverse-genomics-driven capture pipelines also show promise for isolating microbes from complex matrices (127). Culturomics represents a powerful high-throughput approach for investigating human-associated microorganisms. This technique employs diverse incubation conditions to recover heterogeneous microbial populations, followed by taxonomic identification via MALDI-TOF mass spectrometry or 16S rDNA sequencing (125). This approach has uncovered hundreds of previously uncharacterized gut bacterial taxa and revealed functional shifts linked to human lifestyle and industrialization-associated horizontal gene transfer (128, 129). The inaugural culturomics-driven work reported the discovery of multiple previously unknown gut-derived microbes and defined 70 effective incubation conditions for microbial recovery (128). Despite these technical breakthroughs, reliable isolation and cultivation of low-biomass ITM have not yet been achieved, which constitutes a major experimental bottleneck.

#### 2.2.3 Contamination controls

In functional analysis of ITM, contaminant samples originate from multiple sources and severely interferes with result interpretation. A recent study (130) proposed the “RIDE checklist”, a minimum-standard checklist for microbiome research on low-biomass samples, which mandates rigorous controls at sampling, DNA extraction, PCR amplification and other experimental steps. For instance, Nejman (117) established six filtering steps to sequentially remove contaminants derived from DNA extraction, PCR amplification or sequencing runs, negative-control cohorts, paraffin-only blanks used in the study, contaminants introduced during paraffin preparation and storage, and microbial taxa showing no significant enrichment for given tumor types across multiple medical centers, thereby eliminating potential contaminant signals. Collectively, preventing and filtering microbial contamination constitutes a critical procedure for ITM analysis.

## 3 Mechanisms underlying the regulation of intratumoral microbiota immune responses

ITM are increasingly recognized as important modulators of host immune responses and can exert bidirectional effects on antitumor immunity. Emerging evidence indicates that gut-resident microbes can enhance responses to ICI-based immunotherapy by regulating antitumor immunity (131) but may also suppress such immunity (132). Similarly, ITM can shape the TIME and modulate antitumor immunity, thereby influencing cancer progression. These context-dependent effects may either enhance antitumor immunity and immunotherapy efficacy or suppress antitumor immune responses and promote cancer progression.

### 3.1 Intratumoral microorganisms promote and maintain an immunosuppressive microenvironment

#### 3.1.1 Promotion of ROS production

Reactive oxygen species (ROS), metabolic by-products generated through nicotinamide adenine dinucleotide phosphate oxidase activity or mitochondrial respiratory redox reactions, are produced within cells (133). ROS have diverse roles in cancer, including inducing DNA damage, promoting cell proliferation, enabling evasion of apoptosis and anoikis, and inducing tissue invasion and angiogenesis (134). They also contribute to epithelial–mesenchymal transition (EMT), a well-established mechanism of metastasis (135). For instance, *B. fragilis* generates ROS that induce and maintain an tumor immunosuppressive microenvironment, thereby promoting CRC progression (136). *Gastrointestinal Fusobacterium species* similarly promote intestinal carcinogenesis by regulating local inflammation and ROS production (132, 137). Mitochondrial ROS accumulation drives DNA damage and subsequent depletion of NAD^+^ (138), ultimately contributing to the aging of M1-like macrophages. Concurrently, ROS activate matrix metalloproteinases (MMPs), which initiate EMT programs in adjacent epithelial cells. EMT, characterized by loss of epithelial cell polarity and acquisition of mesenchymal traits, subsequently enhances tumor cell invasiveness (139). Furthermore, myeloid-derived suppressor cells (MDSCs) inhibit T-cell function through ROS-dependent production of peroxynitrite (ONOO^-^), a potent oxidant that disrupts T cell signaling and effector functions critical for anti-tumor immunity (140).

#### 3.1.2 Promotion of immune evasion

ITM promote immune evasion by reducing major histocompatibility complex (MHC) molecules expression in tumor cells (141) and upregulating programmed cell death ligand 1(PD-L1) (142), thereby enabling tumor cells to evade cytotoxic immune surveillance. In cervical cancer, *HPV E7* directly upregulated intratumoral surface PD-L1 expression and inhibited cytotoxic T-cell function (143). A bacterium-dependent mechanism of tumor immune evasion involves the Fap2 protein *of F. nucleatum*, which inhibits T- and natural killer (NK) cells activities through TIGIT (144).*F. nucleatum* also binds to and activates the human inhibitory receptor CEACAM1, suppressing immune-cell activity (145). Similarly, an *in vitro* study showed that *H. pylori* binds to CEACAMs through HopQ, thereby inhibiting NK- and T-cell function (146). Mechanistically, the HopQ–CEACAM interaction facilitates translocation of the virulence factor CagA into host cells and promotes IL-8 production (147). Furthermore, *EBV* infection increased IDO expression in B cells, which downregulate NKG2D surface expression in NK cells, suggesting a mechanism by which EBV evades NKG2D-mediated immune attack (148).

#### 3.1.3 Promotion of an immunosuppression microenvironment

Although current research on TME reprogramming aims to identify precise therapeutic targets, accumulating evidence indicates that ITM play a pivotal role in tumor progression by shaping the immunosuppressive TIME. Specifically, ITM can activate or recruit immunosuppressive cell populations, including MDSCs, regulatory T cells (Tregs), neutrophils, and macrophages, thereby inhibiting cytotoxic T-cell function (63). F*. nucleatum* modulates the immunosuppressive TIME by selectively recruiting and accumulating tumor-infiltrating myeloid cells, including CD11b+ myeloid cells, MDSCs, tumor-associated macrophages (TAMs), conventional myeloid dendritic cells (DCs), and CD103+ regulatory DCs, ultimately promoting tumorigenesis (149).Specific microbiota, including *Nevskia ramosa* and *Staphylococcus aureus*, have also been reported to enhance Treg-mediated immunosuppression in the TME and facilitate prostate cancer progression (94). Similarly, Tregs orchestrate the immunosuppressive microenvironment in hepatocellular carcinoma (HCC) associated with *HBV* and *HCV* infection (74, 150). *In vivo* studies have shown that the pancreatic ductal adenocarcinoma (PDAC) microbiome can reshape the TIME by reducing MDSCs, promoting M1 macrophage differentiation, inducing T helper 1 (Th1) differentiation of CD4^+^ T cells, and activating CD8^+^ T-cells (63). Another study demonstrated that the intratumoral fungal mycobiome accelerates the recruitment and activation of Th2 and group 2 innate lymphoid cells (ILC2s) by inducing IL-33 secretion in PDCA *in vivo* and *in vitro (*151). Multi-omics integration also revealed a potential contribution of *Fusobacterium* to the immunosuppressive TME in head and neck squamous cell carcinoma (HNSCC) through modulation of the competing endogenous RNA network and chromatin accessibility (152).

Host transcriptional analyses and tumor immune-infiltration profiling indicate that a high ITM burden reduces CD8 + T-cell infiltration in nasopharyngeal carcinoma, resulting in an immunosuppressive effect (37).

#### 3.1.4 Promotion of inflammatory factors release

Cytokines, including interleukins, interferons (IFNs), tumor necrosis factors (TNFs), chemokines, and growth factors, are central signaling mediators in the TME and coordinate crosstalk among malignant cells, stromal components, and infiltrating immune populations (153). In addition to shaping TME dynamics, these soluble mediators have emerged as versatile therapeutic modalities in cancer immunotherapy (153). F*. nucleatum* can induce the release of inflammatory mediators and has been associated with immunosuppressive responses (144). In a mouse model, microbial products released after disruption of the tumor epithelial barrier activated tumor-infiltrating inflammatory DCs, which induced γδT17-cell polarization. These cells secreted cytokines, including IL-17, IL-8, GM-CSF, and TNF-α, that promoted inflammation and recruited polymorphonuclear MDSCs, converting an inflammatory microenvironment into an immunosuppressive one and promoting CRC progression (154). Commensal bacteria also stimulated MyD88-dependent production of IL-1β and IL-23 by myeloid cells, thereby expanding and activating Vγ6^+^Vδ1^+ γδ^ T cells that produced IL-17 and other cytotoxic effectors, contributing to an immunosuppressive and inflammatory TIME in lung adenocarcinoma (155). Zhu et al. reported that *Lachnoclostridium* may modulate chemokine levels and CD8+ T-cell infiltration, potentially through increased expression of the chemokines CXCL9, CXCL10, and CCL5, and thereby influence survival in patients with cutaneous melanoma (156).

ITM may also modulate antitumor immune responses through the complement system. For example, pathogenic fungi, such as *Malassezia species* can promote PDAC by activating the complement cascade through mannose-binding lectin (62). However, few studies have examined how ITMs influence tumor progression through complement activation. Further research in this area may clarify biological links between microbial ecology and tumor immunology and provide a foundational basis for advancing the clinical translation of microbiota-centric therapeutic approaches.

### 3.2 Intratumoral microorganisms enhance antitumor immune response

#### 3.2.1 Activation of the STING signaling pathway

Studies have shown that the gut microbiome can stimulate stimulator of interferon genes (STING) pathway and induce anticancer immune responses (157). Cyclic adenosine monophosphate produced by *Akmannibacterium mucoproteinae* enhances tumor antigen presentation by activating STING signaling pathway, further inducing macrophage reprogramming and orchestrating the crosstalk between NK cells and DCs, thereby improving the efficacy of ICB in patients with melanoma (157). The intestinal microbe *Bifidobacterium* preferentially colonizes tumor sites and potently stimulates STING/type I interferon (IFN-I) signaling, thereby increasing DC cross-priming after anti-CD47 treatment and providing a mechanism by which ITM may synergize with T-cell-targeted immunotherapy (158).

#### 3.2.2 Promotion of tertiary lymphoid structure maturation

Tertiary lymphoid structures (TLSs) function as localized immune hubs within or adjacent to tumors, orchestrating lymphocyte recruitment, activation, and differentiation to enhance antigen-specific antitumor immunity and support responses to immunotherapy (159). In a mouse CRC model, colonization with *Helicobacter hepaticus* (Hhep) enhanced antitumor immunity by increasing cytotoxic lymphocyte infiltration, inducing Hhep-specific T follicular helper (Tfh) cells, and promoting the maturation of tumor-adjacent TLS. Importantly, adoptive transfer of Hhep-specific CD4^+^ T cells restored anti-tumor effects in Tfh-deficient mice, highlighting that the introduction of immunogenic intestinal bacteria can promote Tfh-associated anti-CRC immunity (160). These findings suggest that ITM can promote TLS maturation and enhance antitumor immune responses; however, this area remains insufficiently studied.

#### 3.2.3 Activation of T- and NK cells

ITM can promote the infiltration and activation of cytotoxic T and NK cells, thereby inducing robust antitumor immune responses (61). In mouse models of esophageal squamous cell carcinoma, *Streptococcus* increased intratumoral CD8+ T-cell infiltration and strengthened antitumor immunity this mechanistic link highlights the potential of leveraging *Streptococcus* to improve the therapeutic effectiveness of anti-PD-1 agents (161). Indole-3-lactic acid, a metabolite derived from *Lactobacillus plantarum*, enhances interleukin-12a (IL-12a) production in DCs by promoting histone H3 lysine 27 acetylation (H3K27ac) at the IL-12a enhancer region. This DC-mediated increase in IL-12a subsequently initiates CD8^+^ T-cell-dependent antitumor immunity and restricts tumor growth (162). Another study revealed that the probiotic *Lactobacillus reuteri* translocates to, colonizes, and persists within melanoma, where its dietary tryptophan catabolite indole-3-aldehyde locally promotes interferon-γ-producing CD8+ T cells and thereby enhances ICI efficacy (163). In a murine CRC model, inosine—a metabolite produced by the bacterium *Bifidobacterium pseudolongum*—induces the expression of Th1-regulating genes in CD4^+^ T cells, a molecular effect that in turn enhances the efficacy of antitumor immunotherapy (164). Rizvi et al. also reported that intratumoral injection of *Bifidobacterium* may activate NK cells, which enhanced tumor regression (19).

#### 3.2.4 Immune activation by bacterial peptides

Intratumoral bacteria commonly localize intracellularly within both tumor and immune cells in the TME. Peptides derived from these bacteria can be processed and presented by antigen-presenting cells(APCs), thereby activating tumor-specific T-cell responses (134). A previous study revealed that melanoma-infiltrating lymphocytes exposed to diverse bacterial peptides exhibited increased IFN-γ secretion, indicating that bacterial peptides presented by tumor cells can elicit T-cell immune reactivity and may serve as targets for tumor-directed therapies (120). Immunofluorescence studies further demonstrated bacterial localization in CD45+ immune cells and tumor cells within the TME, suggesting that ITM-derived peptides may regulate antitumor immunity or influence responses to immunotherapeutic agents (117).

#### 3.2.5 Modulation of the inflammatory microenvironment

Numerous studies have indicated that ITM can activate immune responses and promote antitumor immunity. Neutrophil depletion was associated with increased bacterial abundance and IL-17-mediated tumor-associated inflammatory responses that are essential for CRC growth (50). In addition, *Lactobacillus rhamnosus* strongly promoted anti-tumor immune response, thereby reducing B16 lung metastasis of melanoma in mice (165). Specific *B. bifidum* strains may reduce tumor burden synergistically with PD-1 blockade treatment by enhancing the anti-tumor immune response (166).

ITM modulates antitumor immunity and immunotherapy efficacy either positively or negatively, depending on the microbial species, tumor type, and disease state; however, the factors driving this direction remain unclear. Distinct ITM communities in different tumor tissues can exert different effects on antitumor immune responses (117). Pancreatic cancer has become a major focus of this research because its connection to the digestive tract facilitates microbial colonization that may regulate antitumor immunity. Clarifying the context-dependent effects of ITM on tumor immunity and immunotherapy efficacy is therefore an important direction for future research.

## 4 Microbiota-mediated immunotherapeutic strategies and clinical prospects

### 4.1 Potential application of the microbiome in immunotherapy

Given the established role of ITM in modulating responses to immunotherapy, increasing efforts have focused on manipulating the microbiome for therapeutic purposes. These approaches broadly involve using microorganisms either as direct immunotherapeutic agents or as biological chassis for the delivery of immunotherapeutic payloads (**Figure 2**).

**Figure 2:**
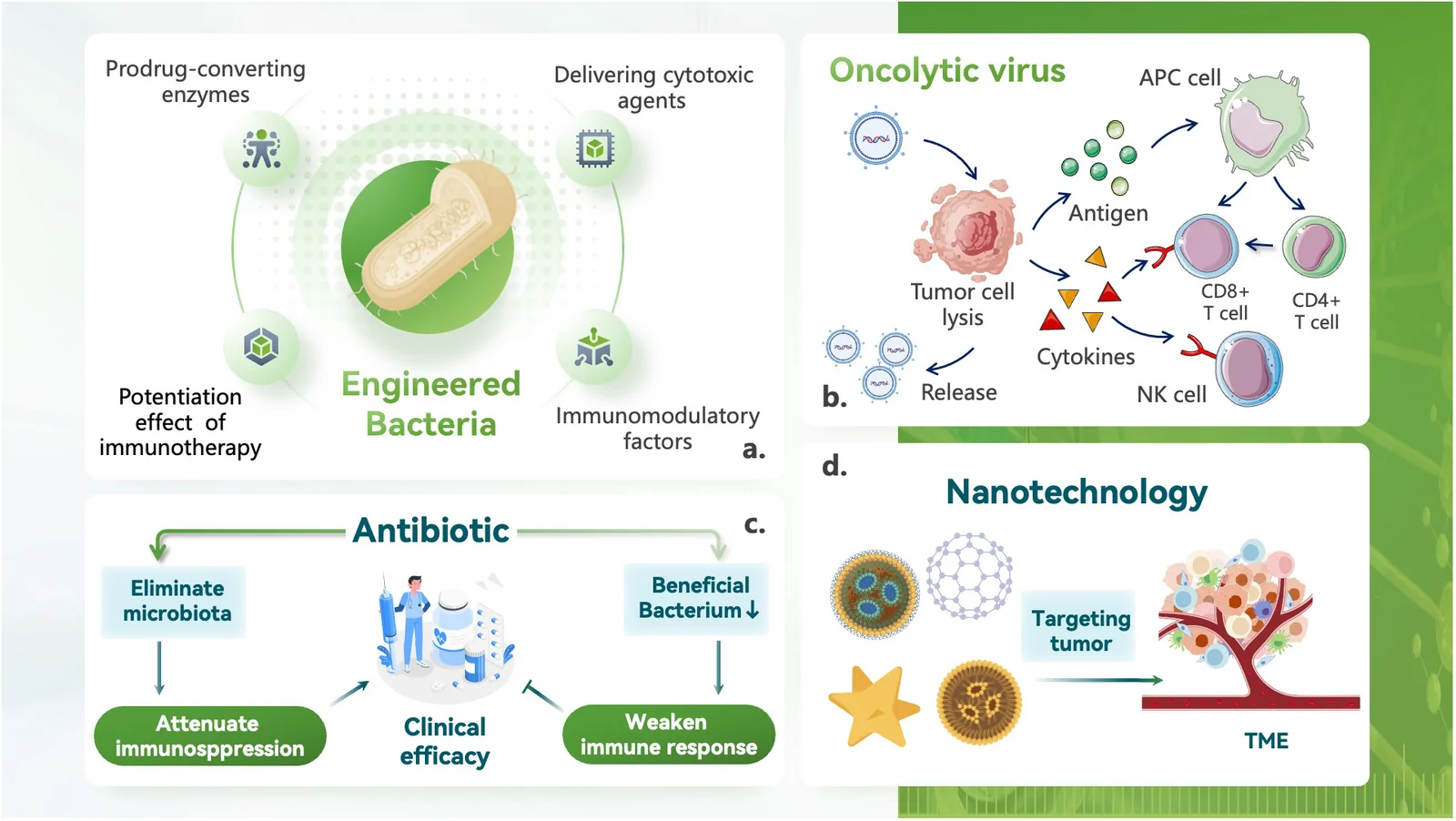
Potential applications of intratumoral microbiota in immunotherapy. **(a)** Engineered bacteria can serve as vectors for delivering cytotoxic agents, prodrug-converting enzymes, and cytokines, thereby enhancing immunotherapeutic efficacy. **(b)** Oncolytic viruses lyse tumor cells, releasing tumor antigens and cytokines that stimulate antitumor immune responses. OVs can also increase PD-L1 expression in tumor and immune cells, potentially increasing sensitivity to anti-PD-L1 therapy. **(c)** Antibiotic targeting of intratumoral microbiota may have both beneficial and detrimental effects. Direct elimination of deleterious intratumoral bacteria with antibiotics represents the most straightforward strategy to target the intratumoral microbiota for improved therapeutic responses. Nevertheless, broad-spectrum antibiotics, while eradicating harmful intratumoral microbes, trigger systemic dysbiosis exemplified by gut microbiota disturbance. They may increase tumor-cell sensitivity to platinum-based chemotherapy, impair the efficacy of immune checkpoint blockade and chimeric antigen receptor-T (CAR-T) cell therapy, and raise the risk of treatment-related adverse events in patients. **(d)** In addition to delivering antibacterial agents that regulate ITM, nanotechnology can remodel the TIME and enhance ICB efficacy. Nanoparticles can precisely target deleterious intratumoral bacterial populations without perturbing microbial community homeostasis within other ecological niches. This advantage is mainly attributed to their robust solid-tumor penetration and favorable tissue-retention capacity. Although relevant research outcomes remain scarce at present, this represents a vital developmental direction for future intratumoral-microbiota-targeted antitumor therapeutics and can substantially enrich existing comprehensive antitumor treatment strategies. Created in BioGDP. https://biogdp.com/zh.

#### 4.1.1 Therapeutic use of engineered bacteria

Genetic engineering can substantially attenuate bacterial virulence, enhance tumor-targeting capacity, and enable the expression of diverse payload-delivery and effector systems. Additionally, engineered bacteria exert modulatory effects on tumor immunotherapy through diverse mechanisms, including the biosynthesis of cytotoxic moieties, production of prodrug-converting enzymes, functioning as immunomodulatory agents, and targeted disruption of the tumor stroma (167).

The use of genetically engineered bacteria, including attenuated *Salmonella*, *Listeria*, and *E. coli* strains, as vectors for tumor-targeted therapy transforms microorganisms from passive components of the TME into active therapeutic platforms. These bacteria can selectively home to and colonize hypoxic and immunosuppressive tumor niches, exploiting the distinctive microenvironment to deliver therapeutic payloads locally (168, 169). Engineered bacteria can secrete cytotoxic agents that directly kill tumor cells, or release immunomodulatory cytokines such as IL-2 and IFN-γ to stimulate antitumor immune responses (170). For instance, engineered *E. coli K-12* could produce cytolysin A (ClyA) to restrain the growth of CRC (171). In another study (172), researchers constructed a repressor-regulated tetracycline efflux system and introduced it into attenuated *Salmonella typhimurium* with inherent tumor-targeting colonization capacity. Upon exogenous tetracycline administration, this system enables bacterial ClyA expression, which allows remote temporal control of bacterial cytotoxic-gene expression for precise tumor therapy. Moreover, engineered *E. coli* can convert ammonia, a metabolic waste accumulated within TME, into L-arginine, thereby potentiating T-cell activity to activate host antitumor immune responses (173). Attenuated *Salmonella typhimurium* secretes *Vibrio vulnificus* flagellar protein B (FlaB). Via the TLR4 signaling pathway, FlaB induces macrophage polarization, elevates the proportion of M1-type macrophages while diminishing M2-type macrophages, and subsequently relieves the immunosuppressive TIME (174).Additionally, engineered bacteria can be designed to degrade extracellular matrix (ECM) components, thereby disrupting the tumor stroma and enhancing immune-cell infiltration and drug penetration (175). Several engineered bacterial therapies have entered clinical trials, underscoring their translational potential and marking a paradigm shift in cancer treatment (169, 176). Furthermore, engineered bacteria can synergize with ICIs by remodeling the TIME and overcoming immunosuppressive barriers (177). However, challenges remain, including ensuring genetic stability, controlling bacterial proliferation, and mitigating safety risks.

Current safety evaluation paradigms predominantly rely on *in vitro* assays and animal models. The FDA has issued guidelines for live biotherapeutic products (LBPs), requiring thorough characterization of strain identity, genetic stability, manufacturing consistency, and clinical safety. Novel approaches, like polymer encapsulation and mucoadhesive microspheres, are being developed to improve bacterial viability, targeted colonization and controllable payload release, optimizing both safety and efficacy. Early-phase clinical trials show acceptable tolerability for LBPs at low-to-moderate doses, with mostly mild, transient adverse events such as diarrhea and fever. For instance, SYNB1891, a genetically engineered tumor-targeting LBP, and CBM588, an unmodified native LBP, have demonstrated such safety profiles (178, 179). Nevertheless, existing clinical evidence is restricted by small cohorts, single-arm designs and short follow-up, making it difficult to identify rare or delayed adverse events and extrapolate results to immunocompromised or comorbid patients. While controlled application with proper monitoring supports the safety of engineered bacterial therapies, larger randomized trials encompassing diverse patient subgroups are required to fully define risk profiles and refine risk-management strategies.

#### 4.1.2 Therapeutic use of oncolytic virus

Oncolytic viruses (OVs) selectively infect and destroy cancer cells and can effectively enhance systemic antitumor immunity. Within the TME, OV-mediated tumor lysis can stimulate the release of inflammatory cytokines and damage-associated molecular patterns and increase the frequency and cytotoxic activity of peripheral NK cells and CD8+ T cells. During tumor lysis, proinflammatory factors and chemokines, including IFNs, TNF-α, IL-12, and IL-6, can activate macrophages, DCs, and T cells through autocrine and paracrine signaling (180).

OV therapy involves administering genetically modified, attenuated viruses that selectively infect tumor cells and induce lysis through multiple mechanisms (181). These viruses are generally engineered for cancer selectivity based on specific genetic alterations, enabling tumor-cell lysis and antigen release, which activate inflammatory responses within the TME and promote antitumor immunity. The oncolytic vaccinia virus *JX-594* can replicate in various tumor cells, and activation of the EGFR/Ras pathway together with inactivation of type I interferon signaling further promotes viral replication and tumor-cell killing (182). *Lymphocytic choriomeningitis virus (LCMV)*, an arenavirus, preferentially replicates in multiple tumors and induces antitumor immunity by recruiting interferon-producing Ly6C^+^ monocytes and enhancing tumor-specific CD8^+^ T cell responses (183). Compared with other clinically evaluated OVs and PD-1 blockade, *LCMV* has shown promising antitumor activity. *IL-23-armed vaccinia virus* can also induce potent antitumor effects and enhance immunotherapy efficacy in multiple mouse tumor models. Studies in CRC and ovarian cancer (184) have demonstrated that oncolytic vaccinia virus enriches T cells within the TME and upregulates PD-L1 expression on tumor cells and T cells. When combined with anti-PD-L1 agents, OVs increase the infiltration of tumor-killing cells and secretion of antitumor cytokines; reduce MDSCs, TAM, and Treg, and terminally-exhausted CD8+ T cells; decrease tumor burden; and prolong survival by activating host antitumor immunity. OVs can further amplify proinflammatory effects by modulating TAM polarization (185). For instance, a genetically engineered *herpes simplex virus type 1 (HSV-1)* expressing OX40 ligand enhanced the proinflammatory phenotype of TAMs and synergized with anti-IL-6 therapy. Combining OVs with ICIs, such as anti-PD-1 and anti-CTLA-4agents, markedly boosted TAM-mediated antitumor activity, underscoring the potential of combination immunotherapy.

In a clinical trial of *SKV-012* for melanoma, three of six patients achieved partial remission, one had stable disease, and two experienced disease progression. No dose-limiting toxicities were observed, and adverse events were mild. These findings indicate that *SKV-012* has a favorable safety profile and promising antitumor activity as an oncolytic virotherapy (186).

OVs may also exploit crosstalk between cancer-associated fibroblasts (CAFs) and cancer cells to penetrate the dense, collagen-rich ECM (187). Cancer cells secrete high levels of fibroblast growth factor 2 (FGF2), which increases their susceptibility to viral infection, and also release TGF-β1, which promotes OV infection of CAFs. This dynamic interplay highlights the therapeutic potential of OVs in overcoming both physical and immunological barriers within TME.

Combining OVs with immunotherapy may provide greater efficacy than either monotherapy. Nevertheless, clinical translation remains limited by the need to establish viral safety, optimize administration and storage, and define parameters such as route, dose, volume, and timing. Although activated TAMs can promote antitumor immunity, they may also clear intratumoral OVs, resulting in compromised therapeutic efficacy. Song et al. (188) identified an interaction between MXRA8 and ZAP that improves the tumor selectivity of oncolytic virus M1; however, OV specificity remains a major concern because some viruses can replicate off target and cause systemic toxicity. Further mechanistic studies are therefore needed to improve biosafety and tumor-targeting precision and to achieve optimal therapeutic effects at lower doses.

#### 4.1.3 Therapeutic use of antibiotics

Direct elimination of deleterious bacteria with antibiotics is a straightforward strategy for targeting ITM and improving responses to cancer treatment. In murine models, aerosolized vancomycin or neomycin reduced pulmonary bacterial abundance, decreased regulatory T cells, and enhanced T- and NK-cell activation (165). This combination of microbial clearance and immunomodulation was accompanied by a marked reduction in lung metastases from B16 melanoma cells. Antibiotics may therefore mitigate the immunosuppressive effects of ITM within the TME. In pancreatic cancer, antibiotic-mediated microbiome manipulation can convert an immunotolerant TME into an immunoactivated state (63). Bernardo et al. (189) reported that oral ampicillin reduced the burden of *Staphylococcus epidermidis* (*S. epidermidis*) in mouse BC tissues and increased microbial populations associated with greater M1 macrophage abundance and fewer MDSCs. The combination of ampicillin and paclitaxel also enhanced the chemotherapeutic effect compared with paclitaxel alone. Similarly, oral antibiotics combined with ICIs may improve immunotherapeutic outcomes (190).

Systemic antibiotics can disrupt immune homeostasis in patients with cancer and produce unintended immunosuppressive consequences, potentially reducing survival and increasing the risk of comorbidities. A prospective multicenter cohort study found that broad-spectrum antibiotic exposure before ICI initiation was associated with poor overall survival in patients with NSCLC, melanoma, and other cancers (191). Another study reported an increased risk of immune-related adverse events (irAEs) after antibiotic treatment (192). Multiple studies (192–195) indicate that although broad-spectrum antibiotics can eliminate harmful intratumoral microbial communities and increase tumor-cell sensitivity to platinum-based chemotherapy, they can also disrupt the intestinal microbiota. Such disruption may impair the efficacy of ICI and chimeric antigen receptor T-cell (CAR-T) therapy and increase treatment-related adverse events.

The regulatory influence of ITM on tumor immunity are bidirectional. This complexity underscores the need for precise delivery systems that selectively target tumor-infiltrating microbiota while minimizing adverse effects on beneficial microbial populations. Nanocarrier-based antibiotic delivery is one potential strategy because it can restrict antibiotic activity to the tumor region. To achieve precise targeting against deleterious intratumoral bacterial populations while preserving microbial community homeostasis, researchers (196) have engineered water-soluble metronidazole-fluorouridine nanoparticles (MTI-FDU) that exhibit potent solid-tumor penetration and favorable tissue retention properties. The metronidazole moiety of these nanoparticles enables targeted elimination of intratumoral *F. nucleatum*. It blocks FadA/E-cadherin/β-catenin and LPS/TLR4/miR-21 signaling cascades activated by *F. nucleatum*, consequently restraining bacterium-driven oncogenesis. Moreover, eradication of *F. nucleatum* by the nanoplatform remodels the TIME and boosts intratumoral infiltration of CD3^+^ and CD8^+^ T-cells. When combined with fluorouridine-mediated cytotoxic effects, the formulation accomplishes dual-targeted delivery of antibacterial and antitumor agents.

#### 4.1.4 Therapeutic use of nanotechnology

Traditional nanomaterials depend on systemic circulation for tumor accumulation and may not penetrate solid tumors beyond the diffusion limit, particularly under hypoxic conditions. Integrating genetic engineering with nanotechnology offers a potential means of improving the efficacy and safety of bacteria-based anticancer therapies. Nanoparticles have valuable physicochemical properties for cancer targeting, diagnosis, and treatment (197) and can support microbiota-directed immunotherapy by enabling targeted delivery of biotherapeutic bacteria and reprogramming the TME (198). First, nanocarriers can deliver antimicrobial agents directly to ITM. For example, intratumoral injection of C. novyi-NT pioneered a novel approach for selectively eliminating tumor tissue (199). Branched gold nanoparticle-coated C. novyi-NT spores have also been injected into tumors (200), where they directly target and kill tumor cells while inducing immune responses and enhancing antitumor efficacy (201). Nanoparticles can be functionalized with surface-modified tumor-targeting ligands, such as folic acid or RGD peptides, to increase tumor-specific accumulation of agents, such as metronidazole and doxycycline and reduce systemic toxicity. For instance, phospholipid nanoparticles cofunctionalized with cRGD peptide and folic acid were developed to deliver doxorubicin to BC cells, substantially increasing drug internalization and cytotoxicity relative to nontargeted or single-ligand systems (202). A biomimetic nanoplatform comprising hybrid membranes derived from engineered T cells and *F. nucleatum* was used to coat nanoparticles loaded with the dual-function agent celastrol. This platform enabled targeted delivery, eliminated *F. nucleatum*, and concurrently blocked the PD-1/PD-L1 checkpoint, thereby increasing immune-cell infiltration and suppressing tumor growth (203). Similarly, in triple-negative breast cancer, outer membrane vesicle-coated nanoparticles delivered metronidazole to eliminate intratumoral *F. nucleatum* while inducing immunogenic cell death through a Fenton-like reaction. The released bacterial components acted as adjuvants and cooperated with the vesicles to promote DCs maturation and T-cell infiltration, converting a cold tumor into an immunologically active tumor and synergizing with immunotherapy (204). Another group (205) constructed an ellagic acid (EA)-endogenous protein (eP) nanocomposite (termed eP-EA) that targets tumors through the enhanced permeability and retention (EPR) effect and eliminates intratumoral bacteria, thereby regulating antitumor immune responses. Such nanomaterials can simultaneously function as drug carriers and antibacterial agents. Nanocarriers can also be engineered to respond to biochemical features of the TME. For example, a “clinic-in-a-dressing” system comprising a hydrogel-nanofiber matrix encapsulates vancomycin within hyaluronic acid-coated zeolitic imidazolate framework nanoparticles, enables pH- and enzyme-responsive release of vancomycin in the acidic, enzyme-rich environment of chronic wounds, which mimics aspects of TME (206). This targeted release maximizes local antibiotic concentrations while reducing off-target effects.

Beyond delivering antibacterial agents to regulate ITM, nanotechnology can remodel the TIME and enhance ICB efficacy. A bioresponsive nanoreactor designed to deplete lactate and normalize tumor vasculature through cascade reactions converted an immunosuppressive phenotype into an immune-activated one, facilitated effector-cell infiltration, and significantly potentiated anti-PD-L1 therapy (207). Another approach uses bacteria-mimetic or bacteria-derived biomimetic nanoparticles. These systems exploit bacterial antigens to stimulate innate immunity, mimicking natural infection and providing a potent danger signal. Intravenously administered bacteria-mimetic gold nanoparticles were phagocytosed by immune cells and delivered to tumors, where photothermal therapy induced tumor damage and enhanced subsequent immune cell recruitment, improving the effects of combined checkpoint blockade (208). Bacterial membrane-coated nanoparticles can also serve as targeted drug delivery systems or immunostimulants. For instance, macrophage membrane-coated nanoparticles loaded with a nitric oxide donor and a TLR7 agonist were designed to target biofilm microenvironments, disrupt bacterial biofilms, and activate innate immunity to eliminate escaping planktonic bacteria. This strategy could potentially be adapted to target ITM and enhance antitumor immunity (209). Bacterial metabolism may also initiate therapeutic cascades. The microbiotic nanomedicine Cu2O@ΔSt colonizes tumors through engineered bacteria and initiates localized reactions that destroy tumor cells and release tumor antigens, thereby sensitizing tumors to subsequent checkpoint blockade and inhibiting metastatic growth (210). Collectively, these nanotechnology-enabled strategies integrate precise antimicrobial activity, targeted ICI delivery, and innate immune activation and may help overcome ITM-mediated immunosuppressive barriers.

The integration of engineered bacteria and nanotechnology represents a paradigm shift in targeted cancer therapy. Bacterial components enable targeted delivery to the tumor niche and can modulate the local immune environment—for instance, by repolarizing TAMs (211).Concurrently, the nanomaterial component provides externally triggerable therapeutic modalities (photothermal, photodynamic, or sonodynamic therapy) or controlled drug release. The integration facilitates hierarchical modulation of the TME combining direct cytotoxicity, immune activation, and microenvironment normalization for a potent, multi-pronged antitumor attack with the potential to minimize systemic side effects through precise spatial and temporal control (211, 212). Bacteria can also be genetically engineered to produce protective surface coatings, such as biofilms, that improve their function as carriers. For example, *E. coli MG1655* was engineered to regulate expression of the Csg gene cluster and produce a biofilm “jacket” (213). This coating enhanced adsorption of therapeutic agents such as the photothermal dye indocyanine green (ICG) and prevented premature drug leakage during systemic circulation, thereby reducing off-target toxicity and improving delivery to inner tumor regions (213). Bacteria can also carry genetic material, such as short hairpin RNA (shRNA) plasmids, to silence specific proteins in tumor cells. An engineered *Salmonella VNP20009* biohybrid system carried CD47 and HSP90 shRNA plasmids intracellularly, reducing expression of these antiphagocytic and protective proteins and enhancing macrophage-mediated phagocytosis (214). Wang et al. developed a DNA-based nanocarrier with antimicrobial and anticancer activity and the ability to regulate the immune microenvironment without evident systemic toxicity (215).

These engineered systems effectively address the dual challenges of achieving deep tumor penetration and maintaining therapeutic agent stability, positioning bacteria as versatile platforms for precision oncology that extend beyond passive nanocarriers (216, 217).

### 4.2 Targeting ITM to enhance immunotherapy efficacy

The interplay between ITM, immunotherapy resistance, and irAEs have received increasing attention. Dysbiosis, particularly within the gut microbiota, is increasingly recognized as a contributor to immunotherapy resistance and a higher incidence of irAEs. The gut microbiome modulates host immunity by influencing immune-cell function, inflammatory signaling, and the TME, thereby affecting both the efficacy and toxicity of immunotherapy. A diverse and balanced gut microbiota enriched in beneficial taxa, including *Bifidobacterium*, *Akkermansia muciniphila*, and *Faecalibacterium prausnitzii*, is associated with stronger antitumor immune responses and better clinical outcomes in patients receiving ICIs (11, 218, 219). Conversely, dysbiosis characterized by reduced diversity and overrepresentation of pathogenic or immunosuppressive bacteria is associated with primary or acquired resistance and an increased risk of irAEs (220, 221). Ongoing and completed clinical trials of microbiome-based interventions combined with immunotherapy are summarized in **Table 2**.

**Table 2:** Clinical trials of intratumoral microbiota combined immunotherapy.

Clinical trails	Experiment time	Phase	Patients	Intervention	Outcome objectives
NCT05462496	2023~2028	Phase II	patients with surgically resectable pancreatic cancer	antibiotics and pembrolizumab	To determine the change in immune activation in pancreatic tumor tissue following treatment with antibiotics, pembrolizumab
NCT05354102	2022~2025	phase I	Patients With NSCLC, Melanoma or RCC	BMC128 in combination with nivolumab	to Evaluate the Safety and Tolerability of BMC128 in Combination With Nivolumab
NCT04909034	2021~2024	—	Patients With NSCLC	MS-20 in Combination with Pembrolizumab	to Evaluate the Safety and Potential Efficacy
NCT01975116	2013∼2017	phase I	patients with recurrent or progressive central nervous system tumors	azurin-derived cell-penetrating peptide p28	stop the growth of tumor cells, either by killing the cells or by stopping them from dividing
NCT04167137	2019∼2021	phase I	participants with advanced/metastatic solid tumors or lymphoma	SYNB1891 monotherapy or in combination with atezolizumab	The primary objective was to evaluate the safety and tolerability of study treatment, with a secondary objective of assessing preliminary tumor response to treatment and exploratory objectives of evaluating the PK/PD of study treatment.
NCT04601402	2020∼2023	phase I/Ib	patients with locally advanced or metastatic solid tumors	GEN-001 and avelumab	to Evaluate the Safety, Tolerability, Biological and Clinical Activities
NCT03775850	2018∼2021	Phase I	Patients With Advanced Metastatic Colorectal Carcinoma, TNBC, and Checkpoint Inhibitor Relapsed Tumors	EDP1503 Alone and in Combination With Pembrolizumab	to assess the safety, tolerability, and efficacy
NCT03637803	2019∼2023	Phase I/II	patients with solid tumors (NSCLC, RCC, bladder cancer or melanoma)	MRx0518 in combination with pembrolizumab	to access Safety And Preliminary Efficacy
NCT03750071	2018~2022	Phase I/II	patients with progressive glioblastoma	VXM01 in combination with avelumab	to evaluate the efficacy and safety
NCT03847519	2019~2022	Phase I/II	Subjects with Metastatic Squamous or NSCLC	ADXS-503 Alone and in Combination with Pembrolizumab	to determine the MTD or MAD and to characterize the preliminary anti-tumor activity
NCT02325557	2015~2020	Phase I/II	participants with metastatic castration-resistant prostate cancer	ADXS31–142 monotherapy and a combination of ADXS31–142 and pembrolizumab (MK-3475)	Dose-Escalation and Safety Study
NCT02002182	2013~2019	Phase II	patients with stage I-IV squamous cell carcinoma of the oropharynx	experimental vaccine: ADXS11-001, uses a live strain of the Listeria monocytogenes (Lm) bacteria	to see if an experimental vaccine, ADXS11-001, is effective in stimulating the body’s defense system against HPV-positive oropharyngeal squamous cell carcinoma before transoral (through the mouth) surgery
NCT00623831	2007~2013	Phase I	subjects with malignant tumors	mixed bacteria vaccine (MBV)	The primary objective was to determine the safety profile of MBV in subjects with malignant tumors that expressed the NY-ESO-1 antigen and to identify the dose that induced the desired pyrogenic effect. Secondary objectives were to evaluate the immunological effects and tumor response of subjects following vaccination.
NCT03190265	2017∼2023	Phase II	Patients With Previously Treated Metastatic Pancreatic Adenocarcinoma	CRS-207, Nivolumab, and Ipilimumab With or Without GVAX Pancreas Vaccine	to study the Safety, Efficacy, and Immune Response
NCT00006254	2000∼2008	Phase I	patients with advanced solid tumors	VNP20009 (nanoparticles)	to stimulate the immune system and stop cancer cells from growing.
NCT00004216	1999∼2008	Phase I	patients with advanced or metastatic solid tumors	VNP20009 (nanoparticles)	to Determine the maximum tolerated dose and safety and the efficacy of VNP20009
NCT00004988	2000∼2002	Phase I	patients with advanced or metastatic cancer	VNP20009 (nanoparticles)	to examine the safety and toxicities of Salmonella bacteria (VNP20009)
NCT01383148	2012∼2016	Phase IIb/III	stage IV NSCLC	first-line therapy combined with TG4010	to compare the efficacy and safety
NCT01924689	2013∼2017	Phase I	patients with treatment-refractory solid tumor malignancies	Clostridium novyi-NT spores (nanoparticles)	to examine the safety
NCT03435952	2018∼2026	Phase I	Patients With Treatment-Refractory Solid Tumors	Pembrolizumab in Combination With Intratumoral Injection of Clostridium Novyi-NT (nanoparticles)	to find the safety and highest tolerable dose of Clostridium novyi-NT spores in combination with pembrolizumab
NCT03265080	2018∼2020	Phase I	participants with select advanced or metastatic solid tumors	ADXS-NEO administered alone and in combination with pembrolizumab	to access Safety And Efficacy

NSCLC, Non-small Cell Lung Cancer; RCC, Renal Cell Carcinoma; PK/PD, pharmacokinetics/pharmacodynamics; TNBC, Triple-negative Breast Cancer; MTD, maximum tolerated dose; MAD, maximum administered dose.

#### 4.2.1 The microbiome improves ICI efficacy

ICIs, including anti-PD-1/PD-L1 and anti-CTLA-4 antibodies, have revolutionized oncology therapy but remain limited by suboptimal response rates and acquired resistance. The microbiome may enhance ICI efficacy by recruiting immune cells. Preclinical and clinical studies indicate that fecal microbiota transplantation (FMT) can restore beneficial microbial communities, promote immune-cell infiltration, and enhance cytotoxic T-lymphocyte function within tumors (222, 223). Tumor-resident microbiota may also contribute to immunotherapy responses. Zheng et al. (224) reported that patients with oral squamous cell carcinoma (OSCC) whose tumors contained higher levels of bacteria of the *genus Peptostreptococcus* had a greater probability of long-term survival. Mechanically, *Peptostreptococcus* drives the recruitment of cytotoxic T cells by activating APCs through the TLR/NLR signaling cascade. Combining *Peptostreptococcus*-based interventions with PD-1 inhibitors increased tumor regression and the density of CD3^+^CD8^+^ T cells within the TME. Additionally, through synthetic biology approaches, Canale et al. (173) constructed an engineered *Escherichia coli Nissle 1917 (EcN)* strain that colonizes tumors and continuously converts intratumoral ammonia into L-arginine, thereby increasing T-cell infiltration and synergizing with PD-L1 inhibition.

Microorganisms may also enhance immunotherapy through immune activation. For instance, oral administration of AHCC^®^, a standardized extract of *Lentinula edodes* mycelia, enhanced the antitumor effects of dual ICB in murine models by modulating gut microbiome composition and increasing CD8+ T cell activity (225). One proposed mechanism of microbiome-mediated augmentation of ICI efficacy is antigenic cross-reactivity between gut bacteria and tumor cells (226). MHC class I-binding epitopes have been identified in enterococcal phage genomes, and these epitopes cross-react with MHC class I-restricted tumor antigens (227). In murine models, enterococcal strains harboring these phages enhanced T-cell-mediated immune responses after chemotherapy or PD-1 inhibition (228). In addition to antigenic cross-reactivity, the microbiome can activate immune cells through specific signaling pathways (158, 164). For instance, butyrate produced by intratumoral microorganisms can activate the PI3K-AKT signaling pathway, promote tumor-cell proliferation and migration, and increase chemoresistance; this mechanism may also the efficacy of immunotherapy (229). Notably, ITM-mediated immune-cell activation may persist after immunotherapeutic treatment (230).

#### 4.2.2 The microbiome improves ACT efficacy

Microbial metabolites have substantial potential in regulating T cell function and enhance adoptive cell therapy (ACT). Short-chain fatty acids (SCFAs) derived from the gut microbiota, including butyrate, can influence T-cell differentiation and function through epigenetic reprogramming (231). As a histone deacetylase inhibitor, butyrate can alter the epigenetic state of T cells and promote expression of memory-associated genes, potentially improving the *in vivo* persistence and antitumor activity of reinfused T cells (232). Indole derivatives may also regulate myeloid-cell function by activating pathways, such as the aryl hydrocarbon receptor, thereby influencing the activity of T cells (233). Certain bacterial components, such as Toll-like receptor (TLR) agonists, can serve as ACT adjuvants. For instance, a strategy combining CpG-loaded liposomes with adoptively transferred CD8+ T cells reduced tumor-infiltrating polymorphonuclear myeloid-derived suppressor cells (PMN-MDSCs) and M2-like macrophages and increased cytotoxic CD8+ T cells in tumors and effector memory CD8+ T cells in the spleen (234). These findings support the use of bacterial components, such as TLR agonists as ACT adjuvants to improve T-cell persistence and function by modulating the local immune environment.

A key strategy for the synergistic treatment of engineered microorganisms with ACT is to use synthetic biology to modify nonpathogenic bacteria, thereby selectively colonizing tumors and expressing immunomodulatory factors *in situ*. Attenuated *Salmonella*, *Listeria*, and other bacteria can be selectively enriched in tumor tissues. In a related delivery strategy, IL-12 tethered to the surface of adoptive T cells through cleavable peptide chains forms a “backpack” that substantially enhances T-cell antitumor activity. IL-12-tethered T cells preferentially increase interferon-γ production and the activity of adoptively transferred T cells at the tumor site while remodeling the immunosuppressive TME, including by converting monocytic MDSCs into inflammatory effector cells. This approach enhances ACT efficacy while limiting systemic toxicity (235). More recent approaches include “sense-and-respond” engineered bacteria that dynamically release therapeutic payloads in response to specific TME signals, enabling precise spatiotemporal coordination with ACT.

#### 4.2.3 The microbiome reduces immunotherapy-related adverse effects

irAEs are associated with alterations in microbiota composition and function. Reduced gut microbial diversity and overgrowth of deleterious bacterial species are closely associated with ICI-induced enteritis. Antibiotic-mediated disruption of the microbiota significantly increases the risk of immune-related enteritis (219, 221).Specific probiotics, like *Akkermansia muciniphila*, may reduce immunotherapy-related adverse effects while enhancing therapeutic efficacy (236). FMT or probiotic supplementation may also alleviate refractory immune-related enteritis by restoring intestinal barrier function and reducing inflammation, thereby decreasing the incidence or severity of immunotherapy-related adverse effects.

#### 4.2.4 Targeted microbiome modulation to support immunotherapy

At present, clinical translational research involving ITM mainly focuses on digestive tract tumors. FMT, probiotics, and prebiotics have emerged as promising strategies for modulating the microbiome and enhancing cancer immunotherapy (**Figure 3**). FMT transfers fecal material from a healthy donor to restore microbial diversity and function and has shown potential for overcoming resistance to ICIs. Preclinical and clinical evidence indicates that FMT can reshape the gut microbiota and improve antitumor immunity by enhancing activation of APCs and cytotoxic T lymphocytes within the TME (237, 238). Nevertheless, widespread clinical implementation of FMT faces multiple challenges. Currently, there are no universal guidelines for donor selection. Significant discrepancies exist across studies with respect to donor age, health status, microbiota composition and screening procedures, which directly compromise the therapeutic consistency and safety of FMT (239). In addition, the standardized operation procedures, optimal administration routes, dosages and long-term safety of FMT still need to be further regulated (240). Therefore, establishing a unified donor screening standard and a consensus on standardized clinical management are the key prerequisites for promoting the application of FMT in clinical practice. Probiotics are live beneficial microorganisms, whereas prebiotics are nondigestible food components that promote the growth of beneficial bacteria; both can modulate the gut microbiome toward an immune-supportive milieu. Specific bacterial taxa, such as *Bifidobacterium* and *Akkermansia muciniphila* have been associated with better responses to ICIs, and probiotic administration has been investigated as a means of improving immunotherapeutic outcomes (241, 242). Whereas, probiotics exhibit extremely low colonization efficiency at tumor sites. Most probiotic strains cannot stably engraft within the intestinal tract, let alone cross the intestinal barrier to reach tumor lesions and exert direct therapeutic effects (243). Furthermore, the therapeutic efficacy of probiotics is modulated by multiple variables including strain specificity, dosage, administration routes and host-related inter-individual variability, leading to substantial heterogeneity in clinical trial outcomes (239, 244). Dietary prebiotics, including natural polysaccharides, may also restore gut microbial balance and regulate the TIME, particularly in CRC models (245). These microbiome-targeted interventions may enhance immune activation and mitigate irAEs by maintaining gut barrier integrity and reducing inflammation (237). Collectively, FMT, probiotics, and prebiotics represent an evolving therapeutic axis for harnessing microbiome-mediated immunomodulation to improve immunotherapy responsiveness.

**Figure 3:**
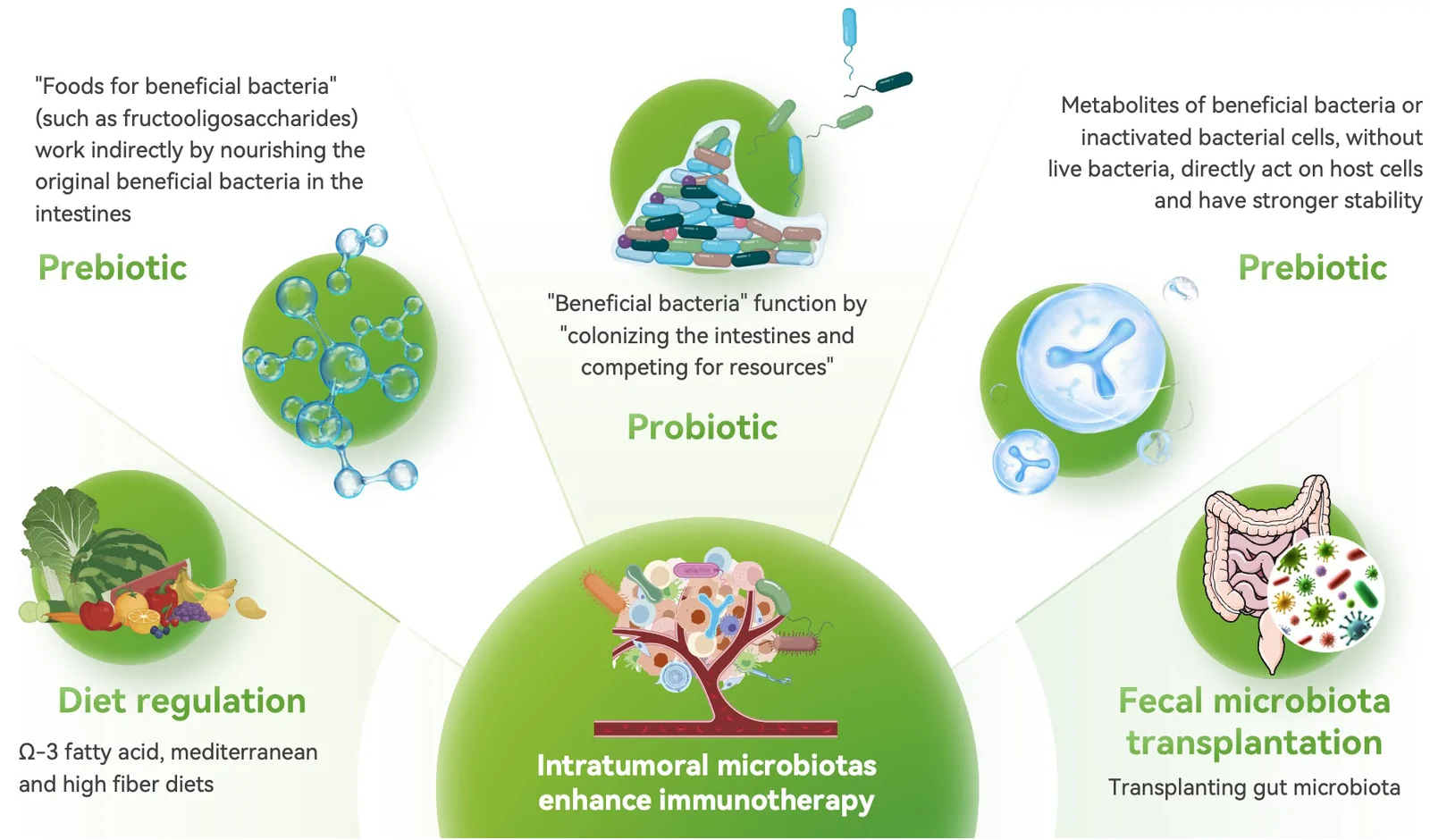
Intratumoral microbiotas enhance immunotherapy. Diet Regulation: The compositional characteristics of gut microbiota are modifiable via nutritional adjustment; omega-3 polyunsaturated fatty acids, Mediterranean diets, and high-fiber diets are documented to confer beneficial effects on immunotherapy responses. Prebiotics: Prebiotics are nutrients for probiotics. Supplementing prebiotics can enhance the activity and quantity of the original probiotics in the intestinal tract, which strengthen the synergistic effect with ICI treatment. Probiotic: the growth of beneficial bacteria can improve dysbiosis effectively and regulate immune function. Such populations are separated from healthy individuals and patients with positive therapeutic responses, while sustaining balanced intestinal microenvironmental conditions (including pH levels and bile acid homeostasis) as well as intact immune homeostasis. Postbiotic: Probiotics indirectly regulate health by the secreting postbiotics (such as SCFA). Even if probiotics fail to colonize successfully, the postbiotics remain effective. Postbiotics regulate immune functions in combination with ICI therapy. FMT: FMT alleviates immunotherapy-associated intestinal microbial disturbance and circumvents ICI therapeutic resistance. Sourced from healthy populations or responders to immunotherapy, this approach reconstructs steady-state gut microbiota composition within the recipient. Created in BioGDP. https://biogdp.com/zh.

Targeted antibiotics have bidirectional effects on the microbiome. While broad-spectrum antibiotics can disrupt microbial diversity and impair immunotherapy efficacy, selective antibiotics targeting deleterious bacterial species within the TME or gut can recalibrate microbial communities to favor antitumor immunity (246).Antibiotics can eliminate pathogenic bacteria and reduce microorganism-mediated immunosuppression; however, broad-spectrum agents may also deplete beneficial microorganisms and diminish immunotherapy efficacy (238, 247). Therefore, targeted antibiotic strategies that avoid indiscriminate microbial depletion are essential for preserving microbiome balance and improving immunotherapy responses.

Nutrition and lifestyle are modifiable factors that influence microbiome composition and immunotherapy efficacy in patients with cancer. Dietary patterns can alter the diversity and function of intestinal microbiota, thereby influencing the activity of the immune system and the therapeutic effect of tumors. Mediterranean, ketogenic, high-fiber diets and intermittent fasting may regulate immune responses, promote anti-tumor immunity, and enhance ICI and other immunotherapy efficacy by altering microbial metabolites, such as SCFAs (248–250). Nutrient availability within the TME also directly affects tumor-cell metabolic reprogramming and immune-cell function. Dietary trace elements and nutrients, such as unsaturated fatty acids, proteins, and amino acids (e.g. methionine and leucine), can influence gut microbial composition and tumor metabolism. Certain dietary components may activate tumor-infiltrating CD8+ T cells and enhance immunotherapy, whereas excessive intake of protein or refined carbohydrates may impair immune surveillance through lactate accumulation and recruitment of immunosuppressive myeloid cells (220, 251, 252). The effects of diet on the gut microbiota are complex and extend beyond fiber content; plant polyphenols and other nutrients also regulate microbial communities and may thereby influence immune responses and tumor progression (253, 254). Besides, identical dietary regimens may trigger markedly distinct microbiota and metabolic responses across individuals. Such heterogeneity mainly stems from complex interactions among host genetic background, baseline microbiota configuration, functional redundancy of gut microbiota, and lifestyle-related factors (255). Herein, to achieve precise and individualized dietary intervention, it is necessary to develop personalized dietary strategies based on microbiota typing (255).

## 5 Discussion: challenges and future directions for intratumoral microbiota in immunotherapy

Heterogeneity of the tumor-associated microbiome and interindividual variability present major challenges to the reproducibility and efficacy of microbiome-informed cancer immunotherapy. Distinct tumor types harbor different microbial communities influenced by anatomical location, the TME, and host factors, resulting in substantial variation among patients. For example, microbial composition differs between right- and left-sided CRC, and these distinct profiles may influence tumor progression and immune responses (45). Similarly, gastric cancer microbiomes differ according to clinicopathological characteristics, including microsatellite instability status and histologic subtype, reflecting complex host–microbe interactions (256). These observations underscore how tumor type and subtype shape microbiome diversity and function and may consequently affect immunotherapy outcomes. Patient-specific factors, including genetics, lifestyle, metabolic status, and medication use, further influence microbiome composition. For instance, histories of smoking and alcohol consumption were associated with greater randomness and instability in the gut microbiome, whereas obesity and hypertension were associated with relatively consistent microbial changes (257).

The molecular mechanisms by which intratumoral microorganisms regulate immunity remain incompletely understood, representing a major limitation of current research. Systematic analyses are needed to determine how microorganisms interact with immune cells through complex molecular networks and how specific signaling molecules, receptors, and metabolites affect the TIME. Intratumoral microorganisms can alter immune-cell activation and infiltration and may regulate immune-checkpoint expression and immune-cell metabolism through the release of microbial metabolites, thereby influencing immunotherapy efficacy (12, 258). The bidirectional interactions between tumor microbiota and host immunity, particularly those underlying immune escape and immunotherapy resistance, also remain insufficiently characterized (259). Technical challenges further complicate this field. First, microbial biomass in tumor tissues is extremely low, creating a substantial risk of environmental contamination and making accurate identification and quantification difficult (260). Second, tumor-associated microbial communities are taxonomically diverse and complex. Although 16S rRNA sequencing supports microbial identification, it provides limited information about microbial function and dynamic interactions with host cells (261). Tumor heterogeneity and TME complexity also limit the ability of any single-omics approach to comprehensively characterize microorganism–immune-cell interactions. Single-cell omics now enables high-resolution analysis of cellular diversity within the TME, but simultaneous high-resolution characterization of microbial components remains at an early stage (262). Spatial omics can colocalize microorganisms and immune cells within tissues and define their spatial distribution, providing important insights into the spatial mechanisms of microbiota-mediated immune regulation (263).

Despite their therapeutic potential, the clinical use of ITM raises important safety concerns. Infection is a major concern because patients with cancer often have impaired immune function. Introducing exogenous or engineered microorganisms may permit uncontrolled growth, dissemination, or translocation in an immunosuppressed host and lead to serious infectious complications. Probiotic-inspired nanovesicles, such as ROMV/TMAO, have shown promise for tumor targeting and immune regulation; however, their safety requires further evaluation to prevent systemic inflammatory responses to bacterial toxins or metabolites (264).
